# Knowledge and stigma of latent tuberculosis infection in Brazil: implications for tuberculosis prevention strategies

**DOI:** 10.1186/s12889-020-09053-1

**Published:** 2020-06-09

**Authors:** Peter F. Rebeiro, Mollie J. Cohen, Heather M. Ewing, Marina Cruvinel Figueiredo, Lauren Saag Peetluk, Kleydson B. Andrade, Marshall Eakin, Elizabeth J. Zechmeister, Timothy R. Sterling

**Affiliations:** 1 Division of Infectious Diseases, Department of Medicine, Vanderbilt University Medical Center, Nashville, TN USA; 2 Vanderbilt Tuberculosis Center, Vanderbilt University Medical Center, Nashville, TN USA; 3 Department of International Affairs, University of Georgia, Athens, Georgia; 4 Vanderbilt University, Nashville, TN USA; 5 Programa Nacional de Controle de Tuberculose ( PNCT), Secretaria de Vigilância em Saúde (SVS), Ministério da Saúde (MS), Brasilia, Brazil; 6 Department of History, Vanderbilt University, Nashville, TN USA; 7 Department of Political Science, Vanderbilt University, Nashville, TN USA

**Keywords:** Tuberculosis, Latent tuberculosis infection, Stigma, Population-based survey, Nationally representative survey, Brazil

## Abstract

**Background:**

Tuberculosis (TB) elimination requires treatment of millions of persons with latent *M. tuberculosis* infection (LTBI). LTBI treatment acceptance depends on population-wide TB knowledge and low stigma, but limited data are available on the relationship between stigma and knowledge. We assessed knowledge of TB disease and LTBI throughout Brazil and examined their association with TB stigma and incidence.

**Methods:**

We performed a nationwide survey with multi-stage probability design through AmericasBarometer from April–May 2017; the sample was representative of Brazil at regional and national levels. Knowledge of and stigma toward TB were assessed by validated survey questions.

**Results:**

Survey-weighted responses of 1532 individuals suggest that 57% of the population knew LTBI can occur, and 90% would seek treatment for it. Regarding active TB, 85% knew TB symptoms, 70% reported they should avoid contact with someone with active TB, and 24% had stigma toward persons with TB (i.e., thought persons with tuberculosis should feel ashamed, or deserved their illness). In regression models adjusting for clinical and demographic variables, knowledge of LTBI was associated with increased stigma toward persons with TB (adjusted odds ratio [OR] = 2.13, 95% confidence interval [CI]: 1·25–3.63, for “should feel ashamed”; OR = 1·82, 95% CI: 1·15–2·89, for “deserve illness”). Adjusting for regional TB incidence did not affect this association.

**Conclusions:**

High proportions of this representative Brazilian population had knowledge of LTBI and were willing to seek treatment for it. However, such knowledge was associated with TB-specific stigma. Strategies to educate and implement treatment of latent tuberculosis must include efforts to decrease TB stigma.

## Background

The global burden of tuberculosis (TB) is enormous, with more than 10 million new cases in 2017 [1]. An estimated 25% of the global population has latent *M. tuberculosis* infection (LTBI) [2], and approximately 80% of active TB cases are due to reactivation of LTBI [3]. Modeling studies show that widespread treatment of LTBI, particularly among those at increased risk of progressing to active TB, could have a profound effect on decreasing global TB burden [4]. Updated 2018 World Health Organization guidelines recommend treatment of LTBI in persons at high TB risk, such as persons living with HIV, children (< 5 years old), household contacts of persons with bacteriologically-confirmed pulmonary TB, and persons with specific underlying medical conditions [5]. Although recommended, treatment rates of LTBI, particularly in countries with high TB burden, have been low [6].

Despite recent advances in its health system [7], Brazil remains among the 30 highest TB-burden countries in the world, with a TB incidence of 33·5–44 cases per 100,000 in 2017 [1, 8]. More than 85% of the Brazilian population lives in urban areas, where TB incidence exceeds the national rate. This high burden highlights the importance of treatment of both active TB and LTBI to decrease TB incidence. Treatment for LTBI has been recommended in Brazil since 1995, however most patients do not start or complete treatment [9] despite screening and treatment being offered free of charge (minimizing the financial impact of these crucial efforts on individuals) [10].

Though reasons for the low rates of diagnosis of LTBI, treatment initiation, and successful completion are multi-factorial [11], stigma associated with TB—including fear of infection—plays an important role [12]. Stigma may reduce willingness to be tested for TB and it may be a barrier to treatment compliance [12, 13]. No universal recommendations for addressing TB stigma exist, though two prior studies suggest improved foundational knowledge may be a key component in reducing stigma and accepting treatment [14].

While some studies have investigated perceptions and experiences with stigma among TB patients and close relatives, there is little research assessing the prevalence of TB-related stigma, and its relationship to disease-specific knowledge, in a general population [12, 15]. We therefore assessed knowledge of TB disease and LTBI throughout Brazil, and its association with TB stigma and incidence. We hypothesized that greater knowledge of active TB disease and LTBI would be associated with lower levels of stigma.

## Methods

### Study population and survey design

We used data from the 2017 AmericasBarometer national survey of Brazil. The Latin American Public Opinion Project (LAPOP) implements the AmericasBarometer biennially, including in 2016–17 in 29 countries in the Americas. In each Latin American country, face-to-face interviews are conducted. The Brazil survey was conducted April 5–May 11, 2017. The sample was generated using a multi-stage probability design, stratified by the country’s five principal regions (North, Northeast, Southeast, South, and Center West), and represents the entire Brazilian population of non-institutionalized voting-age adults. Within each region, the sample was further stratified by municipality size and by urban and rural settings; the probability design carried down to clusters of city blocks, and the study team approached every other house to interview six individuals per cluster.

The AmericasBarometer survey includes approximately 200 questions related to individuals’ experiences, behaviors, and evaluations of their social, economic, and political lives. In addition, the 2017 Brazil survey included 14 questions about TB knowledge, TB health seeking behavior, and stigma/avoidance toward people with TB or Zika virus infection. Though all participants received TB knowledge questions, participants were randomized to receive stigma questions about either TB or Zika, but not both (list of questions and allocation in Supplemental Figure1). The module was designed to capture expressed stigma toward individuals with TB (Zika), with a focus on shame and deservingness due to moral failings, two stigma components which are common [12]. The module also contained questions about avoidance behavior, an effective means to avoid TB transmission, but one often felt and reported by TB patients as stigmatizing [13]. A native Brazilian LAPOP affiliate translated the module into Brazilian Portuguese, and cognitive pre-tests were performed in urban and rural areas of Brazil to test and refine the module. Once deemed valid, the module was programmed into the AmericasBarometer survey, the study team trained, and fieldwork initiated [16]. Further, TB incidence was derived from incident TB cases reported by the National Tuberculosis Programme and population per municipality from the Brazilian Institute of Geography and Statistics (IBGE) website (https://www.ibge.gov.br/en/home-eng.html).

### Study definitions

Participant characteristics were collected with the survey: age (years), gender (interviewer-coded as male or female), self-reported ethnicity (brown (Pardo), black, white, indigenous, or Asian), urban vs. rural residence, self-reported family economic situation (sufficiency of family resources), and self-reported formal education status (none, primary education only, secondary education, or post-secondary education). Participants also reported close friends or family members diagnosed with TB. Additionally, interviewers recorded the participant’s facial skin tone on an 11-point scale (from very light [1] to very dark [11]) [17].

Knowledge of TB symptoms was defined by responses to: 1) knowledge that cough, fever, and chest pain are symptoms of TB; or 2) knowledge that weight loss and night sweats are symptoms of TB. Responses were coded using two separate metrics: responses of “agree” to ≥1 of the TB symptoms questions above, or categorized as “none”, “weight loss/sweating only”, “cough/fever/chest pain only”, or “both.”

Participants were categorized as believing in the effectiveness of non-medical TB treatment if they responded with an incorrect belief (i.e., “very effective”, “effective”, or “neither effective nor ineffective”) to a question regarding the efficacy of alternative/unconventional treatment for TB (herbal or plant-based remedies, home rest, and prayer). Participants who stated there was no cure for TB (unprompted) were included as believing in alternative TB treatments in the analysis. This was due to both responses implying verifiably false beliefs, based on the preponderance of evidence, and therefore indicating poorer TB knowledge.

Knowledge of LTBI was assessed by response to the statement: a person can have TB without feeling or appearing sick. Health-seeking behavior for TB and LTBI were assessed by responses to: 1) If you suspected you had TB, you would seek medical attention, rather than wait for symptoms to disappear; 2) If a doctor or medical professional told you that you may have TB but you do not have symptoms, you would seek medical attention, rather than wait for symptoms to appear. For questions with correct answers (symptoms, health-seeking behavior, treatment effectiveness), participants answering “do not know” were included with the incorrect response, and participants not responding (< 2% for each question) were excluded.

Questions about stigma and avoidance behavior toward TB or Zika virus infection were assessed using two metrics. “Any avoidance” was defined as “agree” to ≥1 of the following: 1) it is important to avoid people who have TB/Zika; 2) if I were on public transport, I would not want to sit next to someone with TB/Zika; 3) If a person were diagnosed with TB/Zika, and shared that information with members of the community, people would avoid contact, even if it means being unfriendly; 4) If a person were diagnosed with TB/Zika, and shared that information with members of the community, people would avoid the person, but be friendly as long as contact was necessary. “Any stigma” was defined by “agree” to ≥1 of the following: 1) people with TB/Zika should feel ashamed of the disease; 2) people who have TB/Zika deserve to be sick because of their immoral behavior [12]. As the avoidance and stigma questions were scalar, we conducted a Cronbach’s alpha analysis to assess their combination or separation; the resulting alpha (0.53) was poor, and their assessment as a singular measure was therefore not justified.

Political knowledge was rated based on the perception of the interviewer on a scale spanning “very high knowledge” to “very low knowledge.” Interviewers were explicitly asked to assess respondents’ knowledge (“conocimiento”), not their sophistication (“sofisticación”). Prior to fieldwork, enumerators received extensive training, which included specific criteria on which to gauge respondents’ political knowledge. These included: their ability to quickly and easily understand questions; their ease and eloquence in discussing political issues; and correct understanding of response scales and options.

### Statistical analysis

All descriptive and regression model analyses were survey-weighted to account for the survey sampling design (described above). Continuous variables were compared by the Kruskal-Wallis test; categorical variables by chi-square statistics. Multivariable and multi-level logistic regression models included covariates determined a priori as known potential confounders of knowledge-stigma relationships in prior TB research*,* and assessed adjusted individual-level associations between participant and community characteristics, stratified by region [18–21]. Age was included in regression models as a continuous covariate using restricted cubic splines with 4 knots, and 40 years (57th percentile) was used as the reference age for ease of interpretation, as it was suitably close to the median (37 years) and mean (38.6 years) ages. Adjusted multi-level logistic models with an outcome of stigma and including an interaction between knowledge of TB symptoms and municipal TB incidence were also fit. Odds ratios (OR) and 95% confidence intervals (CI) or predictive margins were generated from adjusted models to quantify these associations. All analyses were completed using Stata v. 15.1 (StataCorp, LLC, College Station, TX).

## Results

**Fig. 1:**
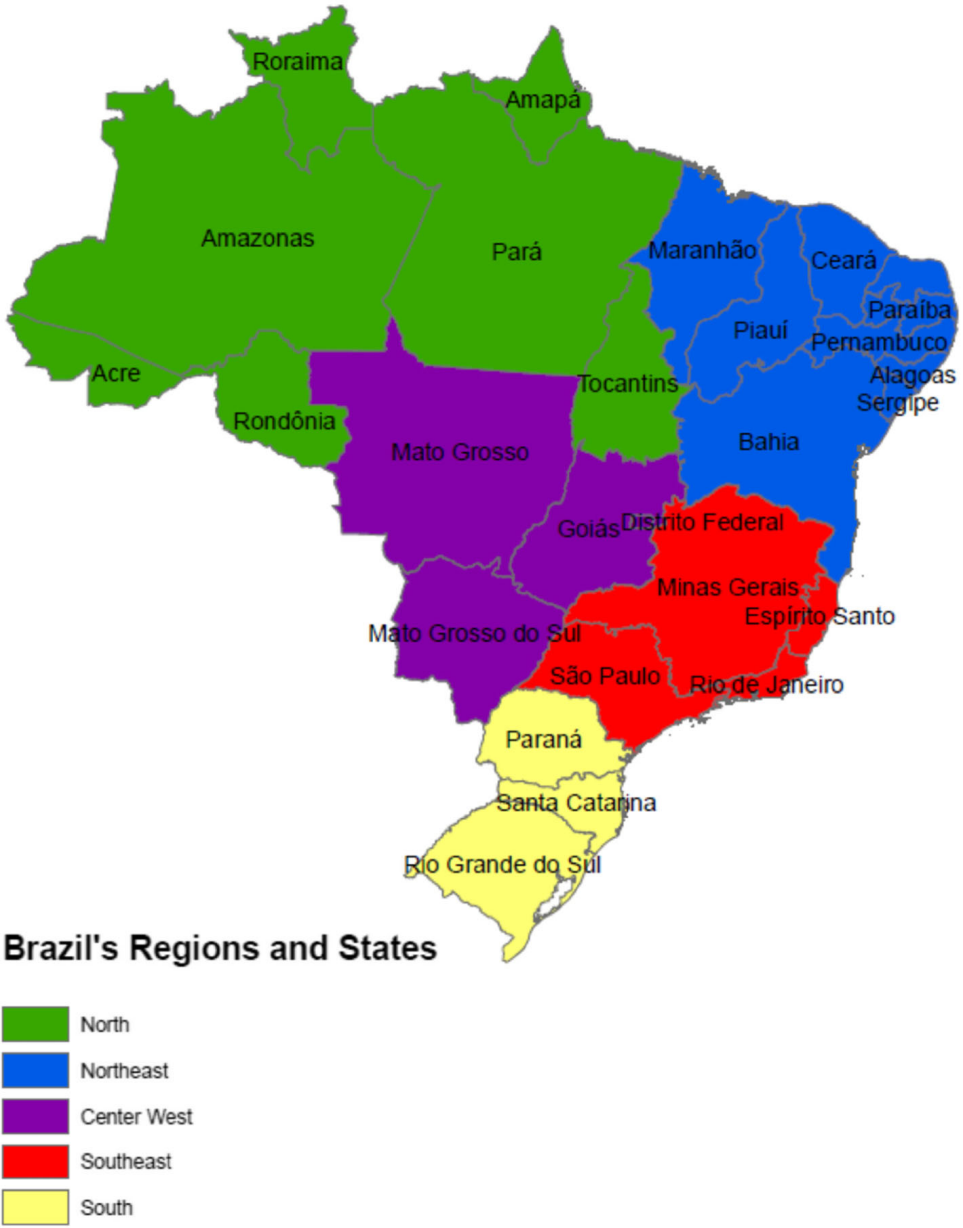
Geography of Brazil, Regions and States

The AmericasBarometer survey and TB- and Zika-specific questions were administered to 1532 persons in Brazil: proportional to the population [22], 1322 respondents in urban areas, and 210 in rural areas [23]. Survey-weighted study population characteristics are in Table 1; regions and states of Brazil are in Fig. 1. TB incidence and sample proportions by geographic units of Brazil in 2016 are in Supplemental Table 1.

**Table 1:** Clinical and demographic characteristics of the study population

Characteristic	North (*n* = 219)	Northeast (*n* = 346)	Southeast (*n* = 491)	South (*n* = 259)	Center West (*n* = 217)	Total (*n* = 1532)	*P*-value
Age, median [IQR]	38.0 [23.0, 48.0]	36.0 [26.0, 51.0]	36.0 [26.0, 49.0]	38.0 [25.0, 53.0]	36.0 [27.0, 48.0]	37.0 [25.0, 49.0]	0.90
Male gender	110 (50.2% [49.4, 51.0])	172 (49.7% [48.7, 50.7])	243 (49.5% [48.8, 50.1])	129 (49.8% [49.1, 50.5])	106 (48.8% [47.0, 50.7])	760 (49.6% [49.2, 50.0])	0.99
Self-reported ethnicity^a^							< 0.001
Brown (Pardo)	123 (56.9% [49.3, 64.3])	161 (46.8% [41.5, 52.2])	191 (39.7% [35.2, 44.4])	57 (22.2% [15.6, 30.6])	110 (51.2% [44.4, 57.9])	642 (41.0% [38.2, 43.8])	
Black	17 (7.9% [4.5, 13.4])	80 (23.3% [17.8, 29.7])	79 (16.4% [12.8, 20.8])	37 (14.4% [9.7, 20.8])	35 (16.3% [12.0, 21.7])	248 (17.4% [15.0, 20.1])	
White	43 (19.9% [15.3, 25.5])	57 (16.6% [12.4, 21.8])	155 (32.2% [27.8, 37.0])	156 (60.7% [52.9, 68.0])	42 (19.5% [14.8, 25.3])	453 (30.5% [27.9, 33.3])	
Indigenous	15 (6.9% [4.3, 11.1])	9 (2.6% [1.3, 5.2])	17 (3.5% [1.9, 6.4])	1 (0.4% [0.1, 2.8])	6 (2.8% [1.4, 5.5])	48 (3.0% [2.1, 4.3])	
Asian	17 (7.9% [5.1, 12.0])	27 (7.8% [6.0, 10.2])	35 (7.3% [5.1, 10.3])	6 (2.3% [1.2, 4.7])	19 (8.8% [5.0, 15.2])	104 (6.8% [5.6, 8.3])	
Urban residence	169 (77.2% [51.6, 91.5])	272 (78.6% [59.1, 90.3])	453 (92.3% [78.1, 97.5])	235 (90.7% [68.4, 97.8])	193 (88.9% [63.6, 97.4])	1322 (87.1% [79.4, 92.1])	< 0.001
Poor Family Economic Situation^b^	122 (56.0% [48.0, 63.6])	215 (63.2% [58.2, 68.0])	250 (51.7% [46.4, 56.8])	113 (44.0% [39.2, 48.9])	97 (46.4% [40.1, 52.8])	797 (53.6% [50.7, 56.4])	< 0.001
Education							0.002
None	2 (0.9% [0.2, 3.5])	12 (3.6% [2.0, 6.5])	10 (2.1% [1.1, 3.8])	0 (0.0)	4 (1.9% [0.8, 4.7])	28 (2.1% [1.4, 3.1])	
Primary	42 (19.3% [14.4, 25.3])	103 (30.9% [26.4, 35.8])	110 (22.7% [18.3, 27.9])	56 (21.8% [16.0, 28.9])	50 (24.3% [17.4, 32.7])	361 (24.6% [22.0, 27.5])	
Secondary	155 (71.1% [65.5, 76.1])	201 (60.4% [55.5, 65.1])	319 (65.9% [61.5, 70.1])	172 (66.9% [59.9, 73.3])	132 (64.1% [55.8, 71.6])	979 (64.8% [62.2, 67.4])	
Post-secondary	19 (8.7% [5.0, 14.7])	17 (5.1% [3.1, 8.4])	45 (9.3% [6.9, 12.5])	29 (11.3% [7.7, 16.2])	20 (9.7% [6.4, 14.5])	130 (8.5% [7.0, 10.2])	
Received TB stigma questions	121 (55.3% [47.6, 62.7])	182 (52.6% [47.3, 57.8])	243 (49.5% [45.1, 53.9])	131 (50.6% [44.9, 56.2])	111 (51.2% [43.3, 59.0])	788 (51.0% [48.4, 53.7])	0.68

Numbers presented as “frequency (weighted column percent within region [95% confidence interval for percent])” unless otherwise indicated

Column percents may not sum to 100 due to rounding

^a^ Excludes “other” race (*n* = 18)

^b^Responses of “Not enough, and having a hard time” or “Not enough, and are stretched”

Knowledge of TB disease and related health-seeking behavior are presented in Supplemental Table 2. Overall, an estimated 85% (95% CI: 83–87) of the population demonstrated knowledge of ≥1 TB symptom, and differences by region were not large. In the sample, 30% (95% CI: 27–33) had a family member or close friend with TB and 52% (95% CI: 49–56) believed in the effectiveness of non-medical TB treatment.

Most of the sample (57, 95% CI: 54–60) knew that LTBI can occur, and such knowledge was somewhat similar by region (Supplemental Table 2). Knowledge of LTBI increased with increasing TB incidence (Supplemental Figure 2). Among the sample, 90% (95% CI: 88–92) would seek healthcare for LTBI. Although most of the sample demonstrated awareness of TB infectiousness, fewer had stigma towards a person with TB (i.e., thought that persons with TB should feel ashamed or deserve their illness; approximately 16% each, and 23% expressed either) (Supplemental Table 3). The prevalence of avoidance behaviors and stigma toward persons with Zika was lower: the percentage of people who would avoid someone with Zika was approximately half that of TB, while the percentage expressing any stigma toward Zika was 16% (95% CI: 14–19) (Supplemental Table 4).

Examining relationships between stigmatizing attitudes for TB, no individual associations were observed (data not shown). Further, neither stigmatizing attitudes, nor advocacy of avoidance of individuals with TB (either by individuals or the community), were associated with TB incidence (data not shown).

In fully adjusted regression models, knowledge of LTBI was associated with an increased risk of stigma towards individuals with TB (i.e., that such persons should feel ashamed, or deserve their illness; OR = 2.13, 95% CI: 1·25–3.63 and OR = 1·82, 95% CI: 1·15–2·89, respectively; Table 2). Such stigma was also independently associated with living in the North region (OR = 2·29, 95% CI: 1·22–4·32 and OR = 2·24, 95% CI: 1·19–4·25, vs. the Southeast, respectively), and having lower education level (OR = 0·56, 95% CI: 0·36–0·87 and OR = 0·60, 95% CI: 0·37–0·96, per unit increase, respectively). In contrast, knowledge of TB symptoms, including fever, chest pain, weight loss, and night sweats (OR = 2.83, 95% CI: 1·61–4.95 vs. no knowledge of symptoms), age of 50 years (OR = 1·30, 95% CI: 1·03–1·65, vs. age 40), and decreased political knowledge (OR = 0·79, 95% CI: 0·67–0·93 per unit increase) were associated with general awareness of TB infectiousness. Though it did not meet a *p* < 0·05 threshold, the association between knowledge of TB symptoms, including fever, chest pain, weight loss, and night sweats, and the expressed stigmatizing attitude that people infected with TB should feel ashamed was noteworthy (OR = 1·71, 95% CI: 0·97–3·02 vs. no knowledge of symptoms).

**Table 2:** Associations between knowledge of TB and avoidance behavior and stigmatizing attitudes toward TB. Survey-weighted odds ratios (and 95% confidence intervals), after adjusting for clinical and demographic factors, including region of Brazil, and stratified by municipality

Characteristic	Avoidance Behavior			Stigmatizing Attitudes	
	Avoid Contact^c^	Any avoidance^d^	Community avoidance^e^	Should Feel Ashamed	Deserve Illness
TB symptom knowledge (vs. none)					
Cough/fever/chest pain only	1.57 (0.88, 2.77)	1.58 (0.91, 2.74)	1.72 (1.00, 2.95)	0.82 (0.42, 1.61)	0.99 (0.46, 2.12)
Weight loss/night sweats only	1.57 (0.65, 3.81)	1.97 (0.74, 5.2)	**2.42 (1.13, 5.20)**	0.61 (0.21, 1.77)	0.32 (0.09, 1.11)
Cough/fever/chest pain and weight loss/night sweats	**2.83 (1.61, 4.95)**	**2.19 (1.28, 3.74)**	**1.89 (1.12, 3.17)**	1.71 (0.97, 3.02)	1.20 (0.59, 2.46)
Knows TB may be latent	1.13 (0.78, 1.62)	1.01 (0.67, 1.54)	1.03 (0.75, 1.42)	**2.13 (1.25, 3.63)**	**1.82 (1.15, 2.89)**
Political knowledge (per unit increase)	**0.79 (0.67, 0.93)**	0.93 (0.75, 1.15)	1.11 (0.91, 1.34)	**0.67 (0.52, 0.86)**	**0.75 (0.59, 0.94)**
Age (vs. 40 years)^a^					
20	0.99 (0.64, 1.54)	0.93 (0.57, 1.54)	0.87 (0.59, 1.28)	1.05 (0.62, 1.80)	**1.90 (1.23, 2.95)**
30	0.78 (0.58, 1.05)	0.85 (0.61, 1.18)	0.82 (0.60, 1.11)	0.84 (0.56, 1.24)	1.08 (0.72, 1.62)
50	**1.30 (1.03, 1.65)**	1.10 (0.84, 1.44)	0.99 (0.78, 1.26)	1.19 (0.86, 1.65)	1.32 (0.91, 1.92)
60	1.38 (0.96, 1.98)	0.97 (0.61, 1.53)	0.68 (0.44, 1.06)	1.17 (0.73, 1.89)	**2.09 (1.28, 3.41)**
Female Gender (vs. Male)	**0.70 (0.51, 0.97)**	0.94 (0.56, 1.55)	1.15 (0.83, 1.60)	0.76 (0.48, 1.20)	0.86 (0.53, 1.41)
Skin tone (per 1-unit increase)	**0.91 (0.83, 1.00)**	0.95 (0.85, 1.06)	0.99 (0.90, 1.09)	1.08 (0.97, 1.22)	1.04 (0.91, 1.18)
Urban residence	1.42 (0.75, 2.66)	0.79 (0.37, 1.69)	**2.35 (1.50, 3.68)**	1.01 (0.56, 1.83)	0.87 (0.48, 1.57)
Family Income (per quintile increase)	1.07 (0.93, 1.23)	1.12 (0.95, 1.33)	1.04 (0.90, 1.20)	0.84 (0.69, 1.02)	0.97 (0.82, 1.16)
Education (per level)^b^	1.09 (0.79, 1.50)	0.77 (0.45, 1.29)	1.43 (0.99, 2.07)	**0.56 (0.36, 0.87)**	**0.60 (0.37, 0.96)**
Region (vs. Southeast)					
North	1.10 (0.71, 1.70)	0.90 (0.55, 1.50)	1.21 (0.79, 1.86)	**2.29 (1.22, 4.32)**	**2.24 (1.19, 4.25)**
Northeast	1.16 (0.73, 1.85)	0.90 (0.54, 1.50)	0.95 (0.62, 1.46)	1.32 (0.68, 2.56)	1.72 (0.95, 3.09)
South	1.49 (0.95, 2.34)	0.65 (0.36, 1.19)	1.09 (0.64, 1.88)	0.95 (0.40, 2.24)	1.08 (0.55, 2.10)
Central-West	1.22 (0.78, 1.93)	1.92 (0.96, 3.87)	1.17 (0.65, 2.11)	1.64 (0.83, 3.24)	1.27 (0.63, 2.55)

**Bold** estimates are statistically significant (*p* < 0.05)

^a^Age was modeled using a restricted cubic spline with 4 knots. The median age of the study population was 37 years and the mean age was 38.6, so 40 years (57th percentile) was used as a reference for ease of interpretation

^b^Per increase in level from none, to primary, to secondary, to post-secondary

^**c**^Response of “agree” to the following statement: If I were on public transport, I would not want to sit next to someone with tuberculosis"

^**d**^Response of “agree” to at least one of the following statements: 1) it is important to avoid people who have tuberculosis; 2) if I were on public transport, I would not want to sit next to someone with tuberculosis; 3) If a person were diagnosed with tuberculosis, and shared that information with members of the community, people would avoid contact, even if it means being unfriendly; 4) If a person were diagnosed with tuberculosis, and shared that information with members of the community, people would avoid the person, but be friendly as long as contact was necessary

^**e**^ Response of “people would avoid contact, even if it means being unfriendly” or “people would avoid the person, but be friendly as long as contact was necessary” to the following statement: if a person were diagnosed with tuberculosis, and shared that information with members of the community

In contrast, when examining the relationship between knowledge of TB symptoms and stigmatizing attitudes toward those with Zika virus, similar models exhibited few strong associations (Supplemental Table 5).

In models adjusting for clinical and demographic factors, including region of Brazil, there were no differences in avoiding contact or stigmatizing attitudes (i.e. TB patients should feel ashamed or deserve their illness), according to TB incidence (Supplemental Table 6). Further, the inclusion of TB symptom knowledge as a potential mediator between incidence and stigma generally exaggerated the strength of association for the attitude that TB patients should feel ashamed and attenuated the association for the attitude that TB patients deserve their illness, but did not alter the results of hypothesis tests.

**Fig. 2:**
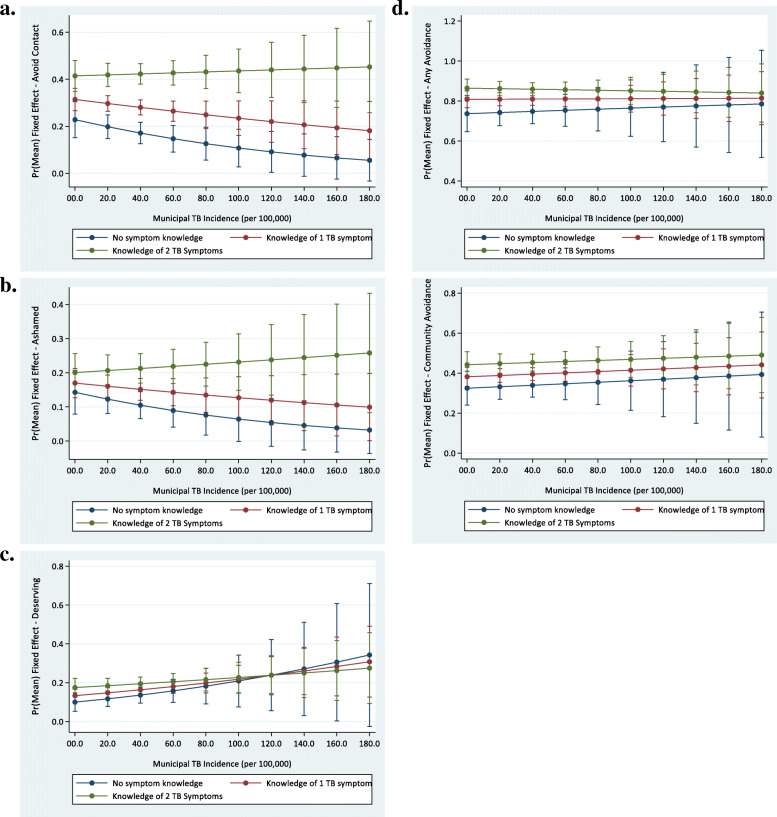
Associations between Avoidance and Stigmatizing Attitudes and Incidence of TB. Stratified by Knowledge of TB Symptoms, from adjusted mixed effects models accounting for clustering at the region level. **a**. Belief that those with TB should avoid contact with others; **b**. Belief that those with TB should feel ashamed; **c**. Belief that those with TB deserve their sickness; **d**. Belief that either the community or the interviewee would avoid contact with someone with TB. The TB incidence scale is the municipal incidence (per 100,000 persons in 2016); minimum value was 0, maximum value was 176.57, median value was 28.33

In adjusted multi-level models accounting for clustering of individuals within region and including an interaction between knowledge of TB symptoms and municipal TB incidence, belief that one should avoid contact with persons with active TB was more likely in persons who had knowledge of TB symptoms than those who did not, and this difference was most pronounced in areas with the highest TB incidence (Fig. 2a). The stigmatizing attitude that persons with TB should feel ashamed was also more likely in persons who had more knowledge of TB (Fig. 2b). However, the stigmatizing attitude that persons with TB deserved their illness was generally not more likely among persons with more knowledge of TB symptoms, particularly in high TB incidence settings (Fig. 2c). Likewise, there was no conditioning relationship for the avoidance measures (Fig. 2d).

## Discussion

In a nationally and regionally representative sample of the population of Brazil, the country with the highest TB burden in the Americas [24], knowledge of TB and LTBI was high. In addition, 90% of the sample said they would seek treatment for LTBI. This bodes well for Brazilian and global efforts to increase the number of persons treated for LTBI [5]. However, contrary to our hypothesis, increased knowledge of LTBI was associated with increased rates of expressed stigma, which could limit TB prevention efforts. To our knowledge, this is one of the first studies to assess awareness of LTBI, knowledge of active TB symptoms, and the relationship among these measures and stigma toward TB in a general, representative population in a high-burden country.

As expected, there was higher knowledge of LTBI among persons living in areas of elevated TB incidence, and living in such areas might decrease belief in the efficacy of alternative (non-traditional) anti-TB treatments. In areas of higher TB incidence, aversion to persons with TB also increased with greater knowledge of TB, which is reasonable given that familiarity with the nature of TB transmission may increase with experience of the disease in everyday life. This did run counter to our initial hypothesis, wherein we assumed that TB-related stigma may be due to a lack of knowledge about TB, and therefore that knowledge of TB would be associated with a decrease in stigma. This was supported by recent work from Datiko, et al., in which persons with higher educational levels, and those with high scores for TB knowledge, had lower stigma scores [25]. However, these findings run contrary to our own, which were derived from a representative population of Brazil, as opposed to a cluster-sampled population in Ethiopia. We also found increased knowledge of TB, whether involving specific symptoms of TB or general knowledge about LTBI, exhibited no association with stigmatizing attitudes toward Zika. This indicates the relationship between TB knowledge and stigma is likely disease-specific, and therefore, interventions for stigma reduction should be specific to TB. In addition, increasing knowledge of TB through education should not increase stigma towards persons with other infectious diseases.

Though the models excluding TB knowledge demonstrated no significant associations between incidence and stigma, our results indicate that knowledge of TB symptoms may partially mediate this relationship in the source and/or target populations.

Our findings may be of particular importance in high-TB burden settings, where social stigma adversely affects persons with TB and the health systems that treat them [12, 26]. In addition, TB often results in poor quality of life, due to both poor physical and mental health [27, 28]. This also affects the perceptions of TB in the general population. It may be the case that improved TB treatment and community knowledge could support each other in a virtuous cycle of diminished stigma and improved individual and population health outcomes. Interventions may not necessarily focus on TB disease education per se, as we found increased probability of stigmatizing attitudes with increasing TB symptom knowledge, but perhaps they should focus on empathy and knowledge of common risk factors for TB.

This study had several limitations. First, stigmatizing attitudes were assessed among individuals who may or may not have been infected with LTBI—in other words, secondary (or expressed) stigma. As there are limited data on valid measures for secondary as opposed to primary (or experienced) TB stigma outside of occupational and household settings, and there are no recognized scales to measure structural or community TB stigma, our own measures of stigmatizing attitudes were not specific to LTBI [29]. However, questions regarding attitudes of community avoidance, shame, and deservingness correspond to expressed stigma scales used for populations living with HIV, which may overlap with TB-affected populations in this setting. Second, though our data were derived from detailed questionnaires, combining rich individual-level data on demographics, socioeconomic status, and knowledge and attitudes regarding TB with population-level data on TB incidence, the potential for unmeasured or residual confounding remains. Finally, we focused on knowledge of TB symptoms as a crucial link between TB incidence and stigma; however, it is possible that knowledge of TB transmission routes and contact patterns may also be related to TB incidence and affect expressed stigma; the elucidation of such relationships remains for future work.

There were several strengths of the study. The AmericasBarometer sampling was broad and regionally representative across the entire nation of Brazil, and survey data covering political, social, economic, and health-specific attitudes were paired with TB incidence data, to allow interpretation of findings alongside TB rates and social context. The focus on knowledge and stigma pertaining to LTBI was also novel in this population. Our findings may have increased relevance and help direct future interventions to improve stigmatizing attitudes toward persons with TB and accelerate treatment of LTBI. We assert that TB programs should take note of these findings as they educate the public, providing education about TB as well as stigma at the same time while exercising care to avoid conveying stigmatizing messages in the process. This foundational knowledge relating political and social awareness to TB knowledge and stigma could also inform the implementation of reforms to improve TB care and outcomes in the Unified Health System in Brazil, which has been historically driven by a politically-engaged civil society and may converge toward universal healthcare [7].

## Conclusions

Patients stigmatized due to public knowledge of their TB diagnosis often become socially isolated, which can substantially reduce quality of life and contribute to negative public health outcomes [12, 27]. Clinics may also struggle to retain patients confronting consequences of social stigmatization. High patient attrition among those receiving treatment for TB in turn increases the risk of drug-resistant TB, which is associated with less favorable outcomes and transmission of drug-resistant TB [12, 26, 28]. The distinction between expressed and experienced stigma may also be of particular importance for policy makers, program funders, and public health officials: education and increased disease-specific knowledge must be provided in ways that mitigate experienced stigma. Expressed stigma itself, however, may not be the major barrier to acceptance of treatment for LTBI in populations similar to Brazil [14], and therefore barriers to treatment acceptance beyond expressed stigma by the general population may need to be addressed simultaneously. Communities across Brazil were knowledgeable about and accepting of treatment for TB and LTBI. However, stigmatizing attitudes toward those with TB disease, and their presence even in settings with high levels of TB knowledge and high TB incidence were also present. This demonstrates that work remains in formulating, funding, and implementing effective education efforts that also mitigate stigma. This is necessary for improving TB and LTBI outcomes, which are key for TB control in Brazil and globally.

## Supplementary information

**Additional file 1: Figure S1**. TB/Zika questions and allocation in AmericasBarometer survey in Brazil. TBQ3 and TBK7A did not have clearly correct answers, so they were excluded from analyses. **Figure S2**. Knowledge of LTBI and incidence of TB in Brazil from adjusted regression models. **Table S1**. Tuberculosis incidence in study survey sites, by region, state, and municipality in Brazil. **Table S2**. Knowledge and health-seeking behavior of tuberculosis disease and latent tuberculosis infection. **Table S3**. Avoidance behavior and stigma towards persons with tuberculosis. **Table S4**. Avoidance behavior and stigma towards persons with zika virus infection. **Table S5**. Associations between knowledge of TB and stigmatizing attitudes toward Zika. Survey-weighted logistic model odds ratios (and 95% confidence intervals), after adjusting for clinical and demographic factors, including region of Brazil, and stratified by municipality. **Table S6**. Evaluating knowledge of TB as a mediator of the influence of TB incidence on avoidance behavior and stigmatizing attitudes toward TB. Survey-weighted odds ratios (and 95% confidence intervals), after adjusting for clinical and demographic factors, including region of Brazil, and stratified by municipality. **Table S7**. Clinical and demographic characteristics of the survey-weighted study population receiving the TB-specific stigma-related questions and Zika-specific stigma-related questions.

## Data Availability

LAPOP survey data are publicly available at the LAPOP website: https://www.vanderbilt.edu/lapop/

## Abbreviations

CI: Confidence interval
HIV: Human Immunodeficiency Virus
LAPOP: Latin American Public Opinion Project
LTBI: Latent *M. tuberculosis* infection
OR: Odds ratio
TB: Tuberculosis
