# Supplementary material for: Knowledge and stigma of latent tuberculosis infection in Brazil: implications for tuberculosis prevention strategies
Source: BMC Public Health. 2020 Jun 9;20:897. doi: 10.1186/s12889-020-09053-1 (PMC7285569; doi:10.1186/s12889-020-09053-1)
Supplement: Supplementary file 1 — Additional file 1: Figure S1. TB/Zika questions and allocation in AmericasBarometer survey in Brazil. TBQ3 and TBK7A did not have clearly correct answers, so they were excluded from analyses. Figure S2. Knowledge of LTBI and incidence of TB in Brazil from adjusted regression models. Table S1. Tuberculosis incidence in study survey sites, by region, state, and municipality in Brazil. Table S2. Knowledge and health-seeking behavior of tuberculosis disease and latent tuberculosis infection. Table S3. Avoidance behavior and stigma towards persons with tuberculosis. Table S4. Avoidance behavior and stigma towards persons with zika virus infection. Table S5. Associations between knowledge of TB and stigmatizing attitudes toward Zika. Survey-weighted logistic model odds ratios (and 95% confidence intervals), after adjusting for clinical and demographic factors, including region of Brazil, and stratified by municipality. Table S6. Evaluating knowledge of TB as a mediator of the influence of TB incidence on avoidance behavior and stigmatizing attitudes toward TB. Survey-weighted odds ratios (and 95% confidence intervals), after adjusting for clinical and demographic factors, including region of Brazil, and stratified by municipality. Table S7. Clinical and demographic characteristics of the survey-weighted study population receiving the TB-specific stigma-related questions and Zika-specific stigma-related questions. [file 12889_2020_9053_MOESM1_ESM.docx]

**Supplemental Figure 1**

TB/Zika questions and allocation in AmericasBarometer survey in Brazil. TBQ3 and TBK7A did not have clearly correct answers, so they were excluded from analyses.

**AmericasBarometer Study Population (N=1532)**

**TB1.** Have you heard of Tuberculosis Yes/No

**TB2.** According to what you know, is it possible for a person to be infected with tuberculosis (TB) without feeling or appearing sick? Yes/No

**TBQ1**. A person is coughing for three weeks and has fever and chest pain. According to what you know, are these symptoms of tuberculosis? Yes/No

**TBQ2**. A person has weight loss and sweating during the night. According to what you know, are these symptoms of tuberculosis? Yes/No

**TBQ3**. A person has digestive problems and stomach pain. According to what you know, are these symptoms of tuberculosis? Yes/No

**HB2A.** If you suspected you had tuberculosis, would you seek medical attention or wait a while to see if the symptoms disappear?

**HB2B.** If a doctor or another medical professional told you that you might have tuberculosis but you didn’t have symptoms, would you be more likely to seek medical care or more likely to wait to see if you developed symptoms first?

**TBK7A.** I'll read you two sentences. Please tell me which one you think fits better about which people develop tuberculosis: 1) People living in dirty and crowded places, such as homeless, drugs, sex workers and prisoners. These are people who are at a high risk of develop tuberculosis. 2) The place where people live or work does not affect the development of tuberculosis. Anyone can develop the disease.

**TBK9.** According to what you know, to what extent unconventional treatments, such as herbal or plant-based remedies, home rest and prayer, can be effective in curing tuberculosis? Very effective/Effective/Neither effective nor ineffective/Ineffective/Very ineffective

**Received tuberculosis stigma questions (N=788)**

**TBS3A.** Suppose that a person in your area was diagnosed with tuberculosis. If this person, when returning home, shared this information with others in the community, do you think people would: 1) avoid contact with this person, even if it meant being unfriendly; 2) avoid contact with this person, but be friendly; 3) offer support for him or her.

**TBS4A.** It is important to avoid contact with people who have tuberculosis not to catch their germs. Agree/Disagree

**TBS4B**. People who have tuberculosis deserve to be sick because of the way they live and their immoral behavior. Agree/Disagree

**TBS4C**. People who have tuberculosis should feel ashamed of the disease. Agree/Disagree

**TBS4D.** If I were on public transport, I would not want to sit next to someone with Tuberculosis. Agree/Disagree

**Received zika stigma questions (N=744)**

**ZIK3A.** Suppose that a person in your area was diagnosed with zika. If this person, when returning home, shared this information with others in the community, do you think people would: 1) avoid contact with this person, even if it meant being unfriendly; 2) avoid contact with this person, but be friendly; 3) offer support for him or her.

**ZIK4A.** It is important to avoid contact with people who have tuberculosis not to catch their germs. Agree/Disagree

**ZIK4B**. People who have zika deserve to be sick because of the way they live and their immoral behavior. Agree/Disagree

**ZIK4C**. People who have zika should feel ashamed of the disease. Agree/Disagree

**ZIK4D.** If I were on public transport, I would not want to sit next to someone with Zika. Agree/Disagree

Randomization

**Supplemental Figure 2**


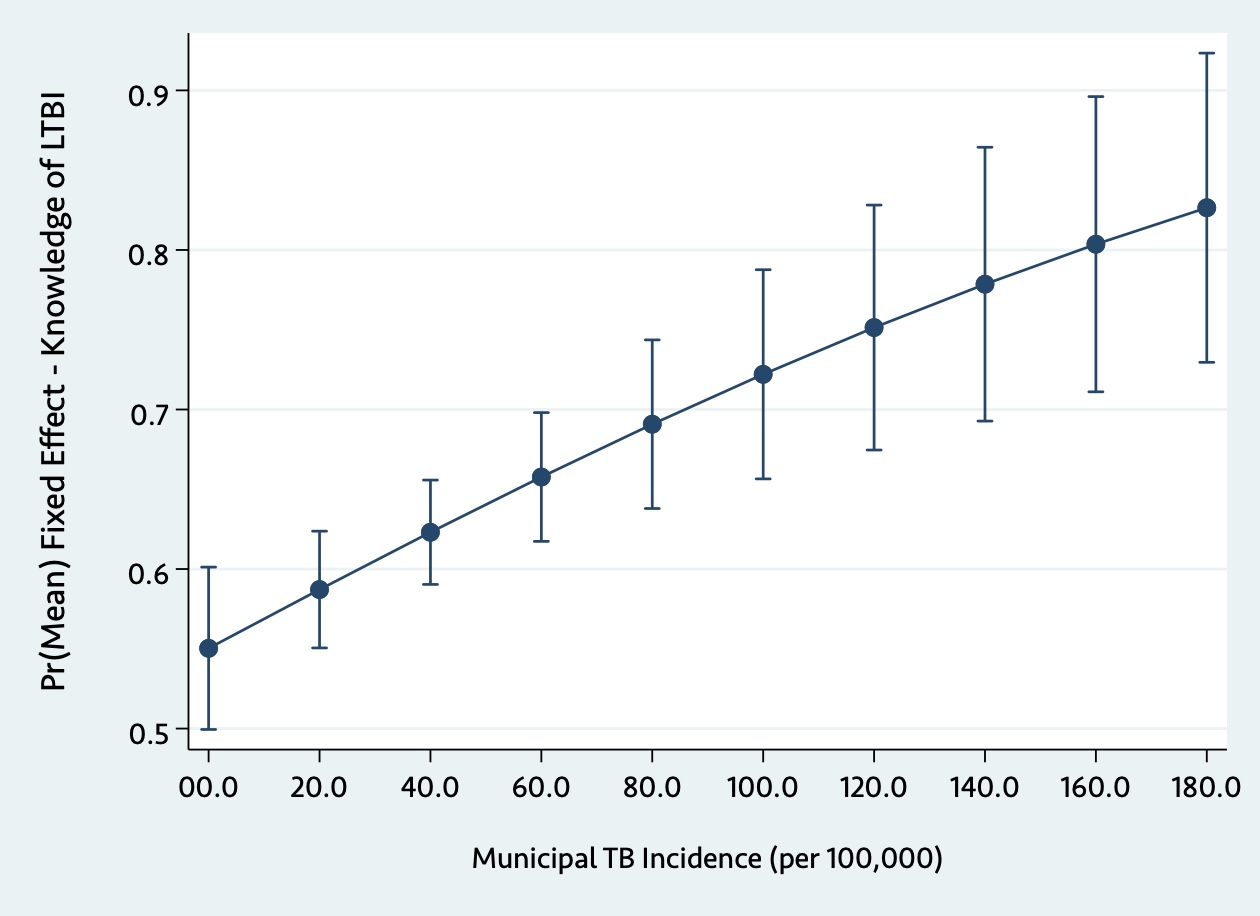
Knowledge of LTBI and incidence of TB in Brazil from adjusted regression models.

**Supplemental Table 1. Tuberculosis incidence in study survey sites, by region, state, and municipality in Brazil.**

| **Region** | **State** | **Municipality** | **Number of TB cases in 2016** | **Estimated population in 2016** | **TB Incidence Rate in 2016 (per 100,000 pop.)** | **Sample proportion (%)** |
| --- | --- | --- | --- | --- | --- | --- |
|  |  |  |  |  |  |  |
| North |  |  | 12,513 | 22,440,941 | 55.8 | 14.30 |
|  | Rondônia |  | 605 | 1,787,279 | 33.9 | 1.56 |
|  |  | Porto Velho | 401 | 511,219 | 78.4 | 0.78 |
|  |  | Buritis | 3 | 38,450 | 7.8 | 0.78 |
|  | Acre |  | 387 | 816,687 | 47.4 | 0.78 |
|  |  | Rio Branco | 285 | 377,057 | 75.6 | 0.78 |
|  | Amazonas |  | 2,718 | 4,001,667 | 67.9 | 4.69 |
|  |  | Codajás | 2 | 27,303 | 7.3 | 0.78 |
|  |  | Itacoatiara | 29 | 98,503 | 29.4 | 0.78 |
|  |  | Manaquiri | - | 29,327 | 0 | 0.78 |
|  |  | Manaus | 2,131 | 2,094,391 | 101.7 | 2.35 |
|  | Roraima |  | 142 | 514,229 | 27.6 | 0.78 |
|  |  | Boa Vista | 137 | 326,419 | 42 | 0.78 |
|  | Pará |  | 3,536 | 8,305,359 | 42.6 | 5.61 |
|  |  | Belém | 1,426 | 1,446,042 | 98.6 | 1.57 |
|  |  | Cametá | 54 | 132,515 | 40.8 | 0.85 |
|  |  | Marabá | 78 | 266,932 | 29.2 | 0.78 |
|  |  | Monte Alegre | 8 | 56,391 | 14.2 | 0.78 |
|  |  | Santana do Araguaia | 2 | 68,934 | 2.9 | 0.85 |
|  |  | Santarém | 116 | 294,447 | 39.4 | 0.78 |
|  | Amapá |  | 244 | 782,295 | 31.2 | 0.85 |
|  |  | Macapá | 209 | 465,495 | 44.9 | 0.85 |
| Northeast |  |  | 25,808 | 66,747,457 | 38.7 | 22.58 |
|  | Maranhão |  | 2,042 | 6,954,036 | 29.4 | 2.41 |
|  |  | Imperatriz | 70 | 253,873 | 27.6 | 0.78 |
|  |  | São Luís | 887 | 1,082,935 | 81.9 | 0.85 |
|  |  | Vitória no Mearin | 14 | 32,161 | 43.5 | 0.78 |
|  | Ceará |  | 3,264 | 8,963,663 | 36.4 | 3.98 |
|  |  | Barreira | - | 20,835 | 0 | 0.78 |
|  |  | Fortaleza | 1,659 | 2,609,716 | 63.6 | 1.57 |
|  |  | Maracanaú | 103 | 223,188 | 46.1 | 0.85 |
|  |  | Pacujá | 3 | 6,186 | 48.5 | 0.78 |
|  | Rio Grande do Norte |  | 950 | 3,474,998 | 27.3 | 1.56 |
|  |  | Parnamirim | 439 | 248,623 | 176.6 | 0.78 |
|  |  | Natal | 65 | 877,662 | 7.4 | 0.78 |
|  | Paraíba |  | 1,101 | 3,999,415 | 27.5 | 3.46 |
|  |  | Alhandra | - | 19,412 | 0 | 0.98 |
|  |  | Conde | - | 24,380 | 0 | 0.85 |
|  |  | João Pessoa | 635 | 801,718 | 79.2 | 0.85 |
|  |  | São Miguel de Taipú | - | 7,131 | 0 | 0.78 |
|  | Pernambuco |  | 4,317 | 9,410,336 | 45.9 | 3.97 |
|  |  | Caruarú | 157 | 351,686 | 44.6 | 0.78 |
|  |  | Jaboatão dos Guararapes | 301 | 691,125 | 43.6 | 0.85 |
|  |  | Petrolina | 85 | 337,683 | 25.2 | 0.78 |
|  |  | Recife | 1,973 | 1,625,583 | 121.4 | 0.78 |
|  |  | Santa Maria da Boa Vista | 6 | 41,475 | 14.5 | 0.78 |
|  | Alagoas |  | 995 | 3,358,963 | 29.6 | 0.78 |
|  |  | Satuaba | 8 | 13,824 | 57.9 | 0.78 |
|  | Sergipe |  | 667 | 2,265,779 | 29.4 | 2.54 |
|  |  | Itabaiana | 35 | 94,393 | 37.1 | 0.91 |
|  |  | Laranjeiras | 8 | 29,418 | 27.2 | 0.85 |
|  |  | Santo Amaro das Brotas | - | 12,086 | 0 | 0.78 |
|  | Bahia |  | 4,197 | 15,276,566 | 27.5 | 3.85 |
|  |  | Feira de Santana | 161 | 622,639 | 25.9 | 0.78 |
|  |  | Riachão do Jacuípe | 1 | 34,715 | 2.9 | 0.72 |
|  |  | Salvador | 1,654 | 2,938,092 | 56.3 | 1.57 |
|  |  | Vera Cruz | 11 | 43,162 | 25.5 | 0.78 |
| Southeast |  |  | 47,644 | 115,042,104 | 41.4 | 32.05 |
|  | Minas Gerais |  | 3,397 | 20,997,560 | 16.2 | 8.72 |
|  |  | Araxá | 6 | 103,287 | 5.8 | 0.78 |
|  |  | Belo Horizonte | 712 | 2,513,451 | 28.3 | 0.78 |
|  |  | Betim | 47 | 422,354 | 11.1 | 0.78 |
|  |  | Divinópolis | 32 | 232,945 | 13.7 | 0.78 |
|  |  | João Pinheiro | 6 | 48,472 | 12.4 | 0.78 |
|  |  | Ouro Fino | 2 | 33,557 | 6 | 0.78 |
|  |  | Pouso Alegre | 31 | 145,535 | 21.3 | 0.78 |
|  |  | Santa Rita do Sapucaí | 6 | 41,886 | 14.3 | 0.78 |
|  |  | Sobrália | 1 | 5,816 | 17.2 | 0.85 |
|  |  | Taiobeiras | 3 | 33,579 | 8.9 | 0.85 |
|  |  | Várzea da Palma | 2 | 38,838 | 5.1 | 0.78 |
|  | Espírito Santo |  | 1,065 | 3,973,697 | 26.8 | 1.56 |
|  |  | Ibatiba | 1 | 25,567 | 3.9 | 0.78 |
|  |  | Vitória | 218 | 359,555 | 60.6 | 0.78 |
|  | Rio de Janeiro |  | 10,615 | 16,635,996 | 63.8 | 7.1 |
|  |  | Belford Roxo | 198 | 494,141 | 40.1 | 0.85 |
|  |  | Duque de Caxias | 678 | 886,917 | 76.4 | 0.78 |
|  |  | Pinheiral | 6 | 24,076 | 24.9 | 0.78 |
|  |  | Queimados | - | 144,525 | 0 | 0.78 |
|  |  | Rio de Janeiro | 6,206 | 6,498,837 | 95.5 | 2.35 |
|  |  | São Pedro da Aldeia | 26 | 98,470 | 26.4 | 0.78 |
|  |  | Volta Redonda | 133 | 263,659 | 50.4 | 0.78 |
|  | São Paulo |  | 16,965 | 44,749,699 | 37.9 | 14.59 |
|  |  | Álvares Machado | 2 | 24,733 | 8.1 | 0.85 |
|  |  | Andradina | 22 | 57,300 | 38.4 | 0.78 |
|  |  | Carapicuíba | 155 | 394,465 | 39.3 | 0.78 |
|  |  | Embu das Artes | 87 | 264,448 | 32.9 | 0.78 |
|  |  | Embu-guacu |  | 67,778 | 128.3 |  |
|  |  | Guarulhos | 502 | 1,337,087 | 37.5 | 0.78 |
|  |  | Indaiatuba | 31 | 235,367 | 13.2 | 0.78 |
|  |  | Ipeúna | - | 7,047 | 0 | 0.78 |
|  |  | Itápolis | 6 | 42,547 | 14.1 | 0.78 |
|  |  | Jandira | 32 | 120,177 | 26.6 | 0.78 |
|  |  | Pindamonhangaba | 32 | 162,327 | 19.7 | 0.78 |
|  |  | São Bernardo do Campo | 241 | 822,242 | 29.3 | 0.78 |
|  |  | São José dos Campos | 158 | 695,992 | 22.7 | 0.78 |
|  |  | São Paulo | 6,020 | 12,038,175 | 50 | 5.16 |
| South |  |  | 10,876 | 35,116,509 | 31.0 | 16.91 |
|  | Paraná |  | 2,123 | 11,242,720 | 18.9 | 4.89 |
|  |  | Alto Periqui | - | 10,253 | 0 | 0.78 |
|  |  | Curitiba | 382 | 1,893,997 | 20.2 | 2.55 |
|  |  | Londrina | 168 | 553,393 | 30.4 | 0.78 |
|  |  | Matelândia | 3 | 17,491 | 17.2 | 0.78 |
|  | Santa Catarina |  | 1,737 | 6,910,553 | 25.1 | 3.12 |
|  |  | Itapema | 7 | 59,147 | 11.8 | 0.78 |
|  |  | Joinville | 197 | 569,645 | 34.6 | 0.78 |
|  |  | São Bento do Sul | 12 | 81,893 | 14.7 | 0.78 |
|  |  | Sombrio | 1 | 29,340 | 3.4 | 0.78 |
|  | Rio Grande do Sul |  | 4,344 | 11,286,500 | 38.5 | 8.85 |
|  |  | Caçapava do Sul | 9 | 34,644 | 26 | 0.78 |
|  |  | Guaíba | 33 | 99,186 | 33.3 | 0.85 |
|  |  | Passo Fundo | 74 | 197,798 | 37.4 | 0.78 |
|  |  | Porto Alegre | 1,558 | 1,481,019 | 105.2 | 1.63 |
|  |  | Santa Maria | 82 | 277,309 | 29.6 | 0.78 |
|  |  | Santana do Livramento | 27 | 82,631 | 32.7 | 0.91 |
|  |  | São Gabriel | 13 | 62,874 | 20.7 | 0.78 |
|  |  | Sapucaia do Sul | 79 | 138,933 | 56.9 | 0.78 |
|  |  | Serafina Correa | 3 | 16,004 | 18.7 | 0.78 |
|  |  | Venâncio Aires | 24 | 71,179 | 33.7 | 0.78 |
| Center West |  |  | 3,576 | 16,651,216 | 21.5 | 14.16 |
|  | Mato Grosso do Sul |  | 837 | 2,682,386 | 31.2 | 2.34 |
|  |  | Aquidauana | 16 | 47,323 | 33.8 | 0.78 |
|  |  | Campo Grande | 269 | 863,982 | 31.1 | 0.78 |
|  |  | Corumbá | 48 | 109,294 | 43.9 | 0.78 |
|  | Mato Grosso |  | 973 | 3,305,531 | 29.4 | 2.41 |
|  |  | Cuiabá | 201 | 585,367 | 34.3 | 0.85 |
|  |  | Nova Brasilândia | - | 3,931 | 0 | 0.78 |
|  |  | Rondonópolis | 68 | 218,899 | 31.1 | 0.78 |
|  | Goiás |  | 800 | 6,695,855 | 11.9 | 7.03 |
|  |  | Aparecida de Goiania | 141 | 532,135 | 26.5 | 0.78 |
|  |  | Campinorte | 2 | 12,198 | 16.4 | 0.78 |
|  |  | Campo Alegre de Goiás | - | 7,024 | 0 | 0.78 |
|  |  | Corumbá de Goiás | 1 | 11,024 | 9.07 | 0.78 |
|  |  | Goianápolis | 2 | 11,460 | 17.5 | 0.78 |
|  |  | Goiania | 212 | 1,448,639 | 14.6 | 1.57 |
|  |  | Leopoldo de Bulhôes | - | 7,758 | 0 | 0.78 |
|  |  | Novo Gama | 6 | 108,410 | 5.5 | 0.78 |
|  | Distrito Federal | Brasília | 270 | 2,977,216 | 9.1 | 2.35 |

Sample proportions may not sum to 1 across regions, across states, or across municipalities due to rounding. Tocantins (North region) and Piaui (Northeast region) were not included in the survey sample. The survey was representative at the region level, not the state level.

**Supplemental Table 2. Knowledge and health-seeking behavior of tuberculosis disease and latent tuberculosis infection.**

| **Focus Area**  **(Sample Population)** | **North**  **(n=219)** | **Northeast**  **(n=346)** | **Southeast**  **(n=491)** | **South**  **(n=259)** | **Center West**  **(n=217)** | **Total**  **(n=1,532)** |
| --- | --- | --- | --- | --- | --- | --- |
| **National weights** | **10,724**  **[10,566- 10,882]** | **41,364**  **[40,387- 42,341]** | **67,408**  **[66,308- 68,508]** | **22,980**  **[22,339-23,621]** | **10,724**  **[10,626-10,822]** | **153,200** |
| Have heard about TB | 9,255  (86.3% [81.1, 90.2]) | 39,451  (95.4% [92.6, 97.1]) | 60,818  (90.2% [86.5, 93.0]) | 21,205  (92.3% [86.8, 95.6]) | 9,093  (84.8% [75.1, 91.1]) | 139,823  (91.3% [89.3, 92.9]) |
| Have a family or friend with TB | 4,260  (39.9% [34.1, 46.0]) | 15,302  (37.2% [31.4, 43.4]) | 18,122  (27.1% [22.4, 32.4]) | 6,211  (27.0% [20.7, 34.5]) | 2,323  (22.0% [15.6, 30.0]) | 46,218  (30.4% [27.4, 33.4]) |
| Would seek treatment for suspected TB | 9,696  (90.4% [84.7, 94.1]) | 37,897  (91.6% [88.2, 94.1]) | 61,779  (91.8% [88.6, 94.2]) | 21,028  (91.5% [87.4, 94.3]) | 9,587  (89.4% [84.1, 93.1]) | 139,987  (91.5% [89.7, 92.9]) |
| Knowledge of at least one main TB symptom^*^ | 9,451  (88.1% [83.5, 91.6]) | 36,582  (88.4% [83.0, 92.3]) | 55,739  (82.7% [79.6, 85.4]) | 19,697  (86.0% [81.2, 89.8]) | 9,093  (85.6% [80.1, 89.8]) | 130,562  (85.3% [83.3, 87.2]) |
| TB symptom knowledge |  |  |  |  |  |  |
| None | 1,273  (11.9% [8.4, 16.5]) | 4,782  (11.6% [7.7, 17.0]) | 11,669  (17.3% [14.6, 20.4]) | 3,194  (14.0% [10.2, 18.8]) | 1,532  (14.4% [10.2, 19.9]) | 22,451  (14.7% [12.8, 16.7]) |
| Cough/fever/chest pain only | 4,162  (38.8% [34.1, 43.7]) | 15,302  (37.0% [31.4, 43.0]) | 24,437  (36.3% [32.7, 40.0]) | 7,808  (34.1% [27.1, 41.9]) | 4,398  (41.4% [34.9, 48.2]) | 56,108  (36.7% [34.1, 39.3]) |
| Weight loss/sweating only | 881  (8.2% [5.7, 11.7]) | 2,511  (6.1% [4.2, 8.7]) | 4,256  (6.3% [4.4, 8.9]) | 1,508  (6.6% [4.6, 9.4]) | 741  (7.0% [4.0, 11.9]) | 9,897  (6.5% [5.3, 7.8]) |
| Both | 4,407  (41.1% [36.0, 46.4]) | 18,769  (45.4% [39.2, 51.7]) | 27,046  (40.1% [35.6, 44.8]) | 10,381  (45.3% [37.0, 54.0]) | 3,954  (37.2% [31.6, 43.2]) | 64,556  (42.2% [39.2, 45.2]) |
| Believe non-medical TB treatment is effective^^^ | 10,724  (53.9% [47.0, 60.6]) | 41,005  (49.3% [43.4, 55.2]) | 66,859  (55.2% [49.9, 60.5]) | 22,714  (50.0% [42.6, 57.4]) | 10,378  (52.4% [43.8, 60.8]) | 151,680  (52.5% [49.4, 55.7]) |
| Knowledge of LTBI^#^ | 6,758  (63.6% [54.8, 71.5]) | 25,464  (61.7% [55.5, 67.6]) | 36,381  (54.1% [48.9, 59.2]) | 12,865  (56.4% [49.8, 62.9]) | 6,079  (57.2% [49.7, 64.4]) | 87,546  (57.4% [54.3, 60.4]) |
| Willingness to seek care for LTBI^§^ | 9,745  (90.9% [87.6, 93.4]) | 38,017  (91.9% [88.7, 94.3]) | 60,544  (89.8% [86.8, 92.2]) | 20,673  (90.0% [83.8, 94.0]) | 9,143  (85.6% [79.5, 90.2]) | 138,121  (90.2% [88.4, 91.7]) |

TB: tuberculosis

LTBI: latent tuberculosis infection

Numbers presented as number (survey-weighted column percent within region [95% confidence interval for percent])

Column percents may not sum to 100 due to rounding

^*^Response of “agree” to at least one of the following statements: 1) coughing for 3 weeks, in addition to fever and chest pain, are symptoms of tuberculosis; 2) weight loss and sweating at night are symptoms of tuberculosis

^^^Response of “very effective”, “effective”, or “neither effective nor ineffective” to the following statement: According to what you know, to what extent are unconventional treatments, such as herbal or plant-based remedies, home rest and prayer, effective in curing tuberculosis?

^#^Response of “agree” to the following statement: A person can have tuberculosis without feeling or appearing sick.

^§^Response of “agree” to the following statement: I would seek medical care if a doctor or healthcare professional told me that I had tuberculosis, even if I did not have symptoms

**Supplemental Table 3. Avoidance behavior and stigma towards persons with tuberculosis.**

| **Focus area** | **North**  **(n=121)** | **Northeast**  **(n=182)** | **Southeast**  **(n=243)** | **South**  **(n=131)** | **Center West**  **(n=111)** | **Total**  **(n=788)** |
| --- | --- | --- | --- | --- | --- | --- |
| **National weights** | **5,925**  **[5,105-6,745]** | **21,758**  **[19,433-24,082]** | **32,400**  **[29,242-35,558]** | **11,002**  **[9,633-12,371]** | **5,436**  **[4,595-6,277]** | **76,521** |
| It is important to avoid someone with TB | 4,113  (69.4% [61.5, 76.3]) | 15,900  (73.1% [65.8, 79.3]) | 22,790  (70.3% [64.9, 75.3]) | 6,477  (58.9% [50.9, 66.4]) | 4,151  (76.4% [67.8, 83.2]) | 53,431  (69.8% [66.5, 73.0]) |
| I would avoid sitting next to someone with TB on public transport | 1,861  (31.4% [25.3, 38.2]) | 6,814  (31.7% [25.3, 38.8]) | 9,885  (30.4% [25.2, 36.2]) | 4,614  (40.6% [32.1, 49.8]) | 1,878  (34.5% [27.8, 42.0]) | 25,051  (32.6% [29.3, 36.1]) |
| Community response to someone with TB: |  |  |  |  |  |  |
| Avoid contact, even if it means being unfriendly | 1,469  (24.8% [17.2, 34.3]) | 4,662  (21.7% [15.6, 29.3]) | 6,452  (19.8% [15.1, 25.6]) | 2,396  (20.8% [14.0, 29.8]) | 1,235  (24.0% [17.5, 32.1]) | 16,215  (21.2% [18.1, 24.6]) |
| Avoid contact, but be friendly | 1,077  (18.2% [14.2, 23.0]) | 3,228  (15.0% [10.2, 21.4]) | 6,727  (20.7% [15.7, 26.8]) | 2,484  (21.5% [15.2, 29.7]) | 988  (19.2% [10.4, 32.8]) | 14,505  (18.9% [16.0, 22.3]) |
| Provide support | 3,379  (57.0% [48.0, 65.6]) | 13,629  (63.3% [55.9, 70.1]) | 19,357  (59.5% [52.3, 66.3]) | 6,654  (57.7% [45.4, 69.1]) | 2,916  (56.7% [46.4, 66.5]) | 45,935  (59.9% [55.7, 64.0]) |
| Any avoidance toward someone with TB^#^ | 4,799  (81.0% [74.1, 86.4]) | 17,813  (81.9% [75.2, 87.0]) | 27,046  (82.8% [78.4, 86.4]) | 8,518  (76.8% [66.7, 84.5]) | 4,794  (89.0% [81.7, 93.6]) | 62,969  (82.0% [79.0, 84.6]) |
| Persons with TB should feel shame | 1,469  (24.8% [20.0, 30.4]) | 4,304  (19.8% [14.7, 26.1]) | 4,256  (12.8% [8.7, 18.4]) | 1,331  (11.5% [5.9%, 21.2]) | 1,137  (20.7% [13.3, 30.8]) | 12,496  (16.0% [13.3, 19.2]) |
| Persons with TB deserve it because of their immoral behavior | 1,420  (24.0% [17.1, 32.6]) | 4,423  (20.9% [15.5, 27.5]) | 3,981  (12.1% [8.9%, 16.2]) | 1,597  (14.0% [9.0%, 20.9]) | 840  (15.9% [9.7%, 25.0]) | 12,262  (16.0% [13.6, 18.7]) |
| Any TB stigma^§^ | 2,106  (35.5% [28.0, 43.9]) | 6,336  (29.6% [23.3, 36.8]) | 6,041  (18.4% [14.0, 23.8]) | 2,129  (18.6% [11.7, 28.3]) | 1,433  (27.1% [19.0, 37.1]) | 18,045  (23.5% [20.4, 26.8]) |

TB: tuberculosis

Numbers presented as number (survey-weighted column percent within region [95% confidence interval for percent])

Column percents may not sum to 100 due to rounding

^#^Response of “agree” to at least one of the following statements: 1) it is important to avoid people who have tuberculosis; 2) if I were on public transport, I would not want to sit next to someone with tuberculosis; 3) If a person were diagnosed with tuberculosis, and shared that information with members of the community, people would avoid contact, even if it means being unfriendly; 4) If a person were diagnosed with tuberculosis, and shared that information with members of the community, people would avoid the person, but be friendly as long as contact was necessary. These elements were combined to reflect a maximum level of avoidance.

^§^Response of “agree” to at least one of the following statements: 1) people with tuberculosis should feel ashamed of the disease; 2) people who have tuberculosis deserve to be sick because of their immoral behavior

**Supplemental Table 4. Avoidance behavior and stigma towards persons with zika virus infection.**

| **Focus area**  **(Sample Population)** | **North**  **(n=98)** | **Northeast**  **(n=164)** | **Southeast**  **(n=248)** | **South**  **(n=128)** | **Center West**  **(n=106)** | **Nationwide**  **(n=744)** |
| --- | --- | --- | --- | --- | --- | --- |
| **National weights** | **4,799**  **[3,986-5,612]** | **19,487**  **[17,302-21,671]** | **33,635**  **[30,796-36,475]** | **11,179**  **[9,857-12,502]** | **5,238**  **[4,410-6,067]** | **74,339** |
| Important to avoid someone with zika | 1,175  (24.5% [14.6, 38.1]) | 6,336  (32.5% [25.5, 40.4]) | 8,375  (24.9% [19.9, 30.7]) | 4,348  (38.9% [30.6, 47.9]) | 1,581  (30.2% [21.8, 40.1]) | 21,815  (29.3% [26.0, 33.0]) |
| Avoid sitting next to someone with zika on public transport | 588  (12.2% [7.3, 19.7]) | 2,989  (15.3% [10.8, 21.3]) | 3,158  (9.4% [6.3, 13.8]) | 2,041  (18.1% [12.4, 25.6]) | 544  (10.5% [6.0, 17.6]) | 9,318  (12.5% [10.3, 15.2]) |
| Community response to someone with zika |  |  |  |  |  |  |
| Avoid contact, even if it means being unfriendly | 539  (11.3% [6.4, 19.4]) | 1,195  (6.2% [3.3, 11.5]) | 2,883  (8.5% [5.1, 13.9]) | 1,508  (13.4% [8.6, 20.3]) | 593  (11.8% [6.7, 19.8]) | 6,719  (9.1% [6.9, 11.8]) |
| Avoid contact, but be friendly | 147  (3.1% [1.0, 9.4%]) | 1,554  (8.1% [4.8, 13.5]) | 3,569  (10.6% [7.5, 14.7]) | 1,775  (15.7% [9.3, 25.3]) | 445  (8.8% [4.7, 15.9]) | 7,490  (10.1% [8.0, 12.7]) |
| Provide support | 4,064  (85.6% [76.8, 91.4]) | 16,378  (85.6% [78.2, 90.8]) | 27,320  (80.9% [75.0, 85.7]) | 7,985  (70.9% [61.1, 79.0]) | 4,003  (79.4% [67.8, 87.6]) | 59,751  (80.8% [77.2, 83.9]) |
| Any avoidance toward someone with zika^#^ | 1,812  (38.1% [27.4, 50.2]) | 8,966  (46.3% [38.9, 53.8]) | 12,768  (38.1% [32.6, 43.9]) | 6,477  (58.4% [49.6, 66.7]) | 2,273  (44.2% [32.8, 56.3]) | 32,296  (43.7% [40.1, 47.4]) |
| Persons with zika should feel shame | 539  (11.2% [6.0, 20.1]) | 3,228  (16.7% [11.9, 22.8]) | 2,883  (8.5% [5.5, 12.9]) | 1,331  (11.7% [7.1, 18.7]) | 593  (11.3% [5.6, 21.5]) | 8,573  (11.5% [9.2, 14.2]) |
| Persons with zika deserve it because of their immoral behavior | 686  (14.6% [8.4, 24.1]) | 2,511  (12.9% [8.8, 18.4]) | 2,746  (8.1% [5.4, 12.0]) | 1,153  (10.2% [6.4, 16.0]) | 642  (12.3% [6.2, 22.7]) | 7,738  (10.4% [8.4, 12.8]) |
| Any zika stigma^§^ | 832  (17.7% [11.1, 27.0]) | 4,304  (22.2% [17.0, 28.6]) | 4,119  (12.2% [8.6, 17.1]) | 1,863  (16.5% [11.6, 23.1]) | 890  (17.0% [9.5, 28.6]) | 12,008  (16.2% [13.6, 19.1]) |

Numbers presented as number (survey-weighted column percent within region [95% confidence interval for percent])

Column percents may not sum to 100 due to rounding

^#^Response of “agree” to at least one of the following statements: 1) it is important to avoid people who have Zika; 2) if I were on public transport, I would not want to sit next to someone with Zika; 3) If a person were diagnosed with Zika, and shared that information with members of the community, people would avoid contact, even if it means being unfriendly; 4) If a person were diagnosed with Zika, and shared that information with members of the community, people would avoid the person, but be friendly as long as contact was necessary

^§^Response of “agree” to at least one of the following statements: 1) people with Zika should feel ashamed of the disease; 2) people who have Zika deserve to be sick because of their immoral behavior

**Supplemental Table 5. Associations between knowledge of TB and stigmatizing attitudes toward Zika.** Survey-weighted logistic model odds ratios (and 95% confidence intervals), after adjusting for clinical and demographic factors, including region of Brazil, and stratified by municipality

| **Characteristic** | **Avoid Contact (Zika)^^^** | **Community avoidance (Zika)^#^** | **Should Feel Ashamed (Zika)** | **Deserve Illness**  **(Zika)** |
| --- | --- | --- | --- | --- |
| TB symptom knowledge (vs. none) |  |  |  |  |
| Cough/ever/chest pain only | 0.59 (0.24, 1.47) | 1.70 (0.71, 4.06) | 1.23 (0.43, 3.56) | 0.72 (0.3, 1.71) |
| Weight loss/night sweats only | 0.82 (0.26, 2.62) | 1.98 (0.72, 5.46) | 0.92 (0.24, 3.59) | **0.17 (0.03, 0.91)** |
| Both sets of symptoms | 0.84 (0.39, 1.81) | 1.50 (0.63, 3.55) | 1.41 (0.51, 3.92) | 1.06 (0.46, 2.43) |
| Knows TB may be asymptomatic | **0.57 (0.34, 0.98)** | **1.55 (1.00, 2.38)** | 1.08 (0.65, 1.80) | 0.76 (0.44, 1.31) |
| Political knowledge (per unit increase) | **0.69 (0.53, 0.90)** | 0.96 (0.75, 1.24) | **0.57 (0.43, 0.77)** | **0.59 (0.43, 0.82)** |
| Age (vs. 40 years)***** |  |  |  |  |
| 20 | **2.45 (1.26, 4.74)** | **1.68 (0.98, 2.88)** | **2.20 (1.16, 4.19)** | 1.48 (0.68, 3.21) |
| 30 | 1.18 (0.76, 1.82) | 1.13 (0.77, 1.66) | 1.04 (0.67, 1.59) | 1.10 (0.66, 1.83) |
| 50 | 1.16 (0.83, 1.63) | 0.92 (0.69, 1.22) | 1.39 (0.92, 2.12) | 1.28 (0.85, 1.92) |
| 60 | 1.47 (0.88, 2.48) | 0.77 (0.50, 1.20) | **2.12 (1.24, 3.64)** | **2.20 (1.37, 3.55)** |
| Female Sex (vs. Male) | **0.41 (0.24, 0.72)** | 0.97 (0.66, 1.43) | **0.32 (0.17, 0.60)** | 0.66 (0.36, 1.21) |
| Skin tone (per 1-unit increase) | **0.82 (0.72, 0.93)** | 1.02 (0.93, 1.13) | 1.03 (0.90, 1.18) | 1.03 (0.92, 1.16) |
| Urban residence | 0.62 (0.31, 1.23) | 1.40 (0.73, 2.70) | 0.56 (0.29, 1.11) | 0.52 (0.27, 1.03) |
| Family Income (per quintile increase) | 1.09 (0.86, 1.38) | 0.96 (0.82, 1.11) | 1.02 (0.83, 1.26) | 1.25 (0.98, 1.60) |
| Education (per level)^§^ | 0.68 (0.43, 1.07) | 1.13 (0.72, 1.77) | **0.59 (0.36, 0.96)** | 0.75 (0.47, 1.19) |
| Region (vs. Southeast) |  |  |  |  |
| North | 1.45 (0.64, 3.31) | 0.67 (0.33, 1.39) | 1.42 (0.56, 3.55) | 2.25 (0.96, 5.27) |
| Northeast | 1.49 (0.75, 2.97) | 0.72 (0.38, 1.39) | 1.86 (0.96, 3.60) | 1.47 (0.69, 3.11) |
| South | 1.77 (0.90, 3.49) | **1.84 (1.04, 3.26)** | 1.33 (0.58, 3.09) | 1.07 (0.51, 2.27) |
| Center West | 1.36 (0.62, 2.97) | 0.88 (0.47, 1.64) | 1.27 (0.50, 3.26) | 1.54 (0.63, 3.80) |

**Supplemental Table 6. Evaluating knowledge of TB as a mediator of the influence of TB incidence on avoidance behavior and stigmatizing attitudes toward TB.** Survey-weighted odds ratios (and 95% confidence intervals), after adjusting for clinical and demographic factors, including region of Brazil, and stratified by municipality.

| **Characteristic** | **Avoid Contact^^^** | | **Any avoidance^#^** | | **Community avoidance^%^** | | **Should Feel Ashamed** | | **Deserve Illness** | |
| --- | --- | --- | --- | --- | --- | --- | --- | --- | --- | --- |
|  | **Excluding TB Knowledge** | **Including TB Knowledge** | **Excluding TB Knowledge** | **Including TB Knowledge** | **Excluding TB Knowledge** | **Including TB Knowledge** | **Excluding TB Knowledge** | **Including TB Knowledge** | **Excluding TB Knowledge** | **Including TB Knowledge** |
| TB Incidence^!^ (vs. <10) |  |  |  |  |  |  |  |  |  |  |
| 10 to <50 | 1.32 | 1.30 | 1.26 | 1.28 | 1.32 | 1.30 | 0.66 | 0.64 | 1.37 | 1.33 |
|  | (0.90, 1.94) | (0.86, 1.97) | (0.77, 2.06) | (0.77, 2.13) | (0.82, 2.11) | (0.80, 2.12) | (0.36, 1.23) | (0.33, 1.25) | (0.72, 2.59) | (0.67, 2.62) |
| 50 to <100 | 1.07 | 0.97 | 1.02 | 1.01 | 1.04 | 1.02 | 0.81 | 0.72 | 1.28 | 1.16 |
|  | (0.64, 1.79) | (0.58, 1.61) | (0.54, 1.92) | (0.54, 1.88) | (0.58, 1.85) | (0.57, 1.83) | (0.35, 1.88) | (0.30, 1.74) | (0.59, 2.77) | (0.52, 2.61) |
| ≥100 | 1.17 | 0.97 | 1.64 | 1.50 | 1.53 | 1.40 | 1.10 | 0.90 | 2.43 | 2.20 |
|  | (0.64, 2.14) | (0.53, 1.76) | (0.77, 3.49) | (0.71, 3.17) | (0.69, 3.37) | (0.64, 3.08) | (0.25, 4.9) | (0.19, 4.19) | (0.73, 8.03) | (0.63, 7.72) |
| TB symptom knowledge (vs. none) |  |  |  |  |  |  |  |  |  |  |
| Cough/fever/chest pain only |  | 1.54 |  | 1.55 |  | 1.69 |  | 0.85 |  | 0.97 |
|  |  | (0.86, 2.73) |  | (0.88, 2.73) |  | (0.98, 2.91) |  | (0.43, 1.66) |  | (0.45, 2.12) |
| Weight loss/night sweats only |  | 1.5 |  | 1.86 |  | 2.3 |  | 0.63 |  | 0.31 |
|  |  | (0.62, 3.66) |  | (0.68, 5.06) |  | (1.05, 5.02) |  | (0.22, 1.8) |  | (0.09, 1.08) |
| Both sets of symptoms |  | **2.86** |  | **2.17** |  | **1.88** |  | 1.72 |  | 1.18 |
|  |  | **(1.63, 5.02)** |  | **(1.27, 3.7)** |  | **(1.11, 3.17)** |  | (0.97, 3.04) |  | (0.57, 2.45) |
| Knows TB can be asymptomatic |  | 1.13 |  | 1.01 |  | 1.02 |  | **2.18** |  | **1.79** |
|  |  | (0.78, 1.62) |  | (0.66, 1.53) |  | (0.74, 1.41) |  | **(1.27, 3.77)** |  | **(1.12, 2.86)** |

All models are adjusted for political knowledge, age, sex, skin color, urban residence, family income, education, and region

**Bold** estimates are statistically significant (p<0.05)

! per 100,000 population

*****Age was modeled using a restricted cubic spline with 4 knots.

^^^Response of “agree” to the following statement: If I were on public transport, I would not want to sit next to someone with tuberculosis”

^#^Response of “agree” to at least one of the following statements: 1) it is important to avoid people who have tuberculosis; 2) if I were on public transport, I would not want to sit next to someone with tuberculosis; 3) If a person were diagnosed with tuberculosis, and shared that information with members of the community, people would avoid contact, even if it means being unfriendly; 4) If a person were diagnosed with tuberculosis, and shared that information with members of the community, people would avoid the person, but be friendly as long as contact was necessary

^%^ Response of “people would avoid contact, even if it means being unfriendly” or “people would avoid the person, but be friendly as long as contact was necessary” to the following statement: if a person were diagnosed with tuberculosis, and shared that information with members of the community.

**Supplemental Table 7.** **Clinical and demographic characteristics of the survey-weighted study population receiving the TB-specific stigma-related questions and Zika-specific stigma-related questions.**

| **Characteristic** | **TB-specific stigma questions** | **Zika-specific stigma questions** |
| --- | --- | --- |
| Sample population | 788 | 744 |
| National weights | 78,152  [73, 778 - 82,527] | 75, 048  [71,142 - 78,953] |
| Age, mean [95% CI] | 38.52 [37.63-39.41] | 38.61 [37.58-39.63] |
| Male sex | 40,433 (51.7% [49.3, 54.2]) | 35,561 (47.4% [44.8, 50.0]) |
| Self-reported ethnicity^^^ |  |  |
| Brown (Pardo) | 31,986 (41.5% [37.4, 45.7]) | 30,000 (40.5% [37.1, 44.0]) |
| Black | 13,038 (16.9% [14.0, 20.2]) | 13,217 (17.8% [14.7, 21.5]) |
| White | 23,430 (30.4% [26.7, 34.3]) | 22,686 (30.6% [27.2, 34.3]) |
| Indigenous | 2,690 (3.5% [2.1, 5.8]) | 1,840 (2.5% [1.5, 4.2]) |
| Asian | 4,876 (6.3% [4.7, 8.5]) | 5,460 (7.4% [5.7, 9.5]) |
| Urban residence | 67,296 (86.1% [77.4, 91.8]) | 66,077 (88.0% [80.7, 92.9]) |
| Poor Family Economic Situation* | 41,767 (54.4% [50.6, 58.2]) | 39,052 (52.7% [48.9, 56.4]) |
| Education |  |  |
| None | 1,653 (2.2% [1.2, 3.9]) | 1,450 (2.0% [1.1, 3.5]) |
| Primary | 18,034 (23.6% [20.1, 27.5]) | 18,878 (25.7% [22.5, 29.1]) |
| Secondary | 50,526 (66.2% [62.1, 70.0]) | 46,673 (63.5% [60.4, 66.4]) |
| Post-secondary | 6,149 (8.1% [6.2%, 10.4]) | 6,553 (8.9% [7.0, 11.3]) |

Numbers presented as number (survey-weighted column percent [95% confidence interval for percent])

Column percents may not sum to 100 due to rounding

^ Excludes “other” race (n=18)

*Responses of “Not enough, and having a hard time” or “Not enough, and are stretched”
